# Compliance with NPIs and possible deleterious effects on mitigation of an epidemic outbreak

**DOI:** 10.1016/j.idm.2021.06.001

**Published:** 2021-07-12

**Authors:** Maria Vittoria Barbarossa, Jan Fuhrmann

**Affiliations:** a Frankfurt Institute for Advanced Studies, Frankfurt, Germany; b Jülich Supercomputing Centre, Forschungszentrum Jülich, Jülich, Germany

**Keywords:** Non-pharmaceutical intervention, Epidemic model, Compliance, Reproduction number, COVID-19

## Abstract

The first attempt to control and mitigate an epidemic outbreak caused by a previously unknown virus occurs primarily via non-pharmaceutical interventions (NPIs). In case of the SARS-CoV-2 virus, which since the early days of 2020 caused the COVID-19 pandemic, NPIs aimed at reducing transmission-enabling contacts between individuals. The effectiveness of contact reduction measures directly correlates with the number of individuals adhering to such measures. Here, we illustrate by means of a very simple compartmental model how partial noncompliance with NPIs can prevent these from stopping the spread of an epidemic.

## 1 Introduction

Faced with an epidemic outbreak caused by a previously unknown virus, effective medication or vaccines are usually not available. Mitigation therefore tends to primarily rely on non-pharmaceutical interventions (NPIs). This was no different in case of the SARS-CoV-2 virus, which has caused the COVID-19 pandemic. Most countries applied NPIs aimed at reducing contacts between infectious and susceptible individuals. Such measures range from social distancing or school closure to most severe lockdown periods. Reducing most contacts between individuals, NPIs necessarily reduce epidemiologically relevant contacts, or *effective contacts*, viz., those between infectious and susceptible individuals during which the virus is successfully transmitted. The effectiveness of contact reduction measures directly correlates with the number of individuals actually adhering to the measures(Acuña-Zegarra et al., 2020). Is it better to have stricter measures followed by a small fraction of the population or almost universally adopted moderate measures? There is an obvious trade-off. Stricter measures are more effective in reducing contacts between compliant individuals but are also less likely to be realistically applicable to many people. Here, we illustrate by means of a very simple mathematical model how partial noncompliance with NPIs can prevent them from stopping the spread of an epidemic. Though the working example is the COVID-19 epidemic, specifically in Germany in late summer/early fall of 2020, the model is generally valid and flexible to be applied to other infectious diseases.

## 2 Methods

The core of the model used in this note extends the known *S-E-I-R* (susceptibles–exposed–infected–recovered) model for disease dynamics (Brauer et al., 2019). The ordinary differential equations (ODEs) approach that we use assumes that the population is homogeneous and well-mixed within a region. Individuals are classified according to their status with respect to the virus spread in the community. Susceptible individuals (*S*) can be infected. The time between exposure to the virus (becoming infected) and symptom onset, commonly known as “exposed phase” or incubation period is divided in three stages (*E*_*j*_, *j* = 1, 2, 3), the last being presymptomatic and contagious. Besides allowing for the presymptomatic infectious period to be fixed as the last stage of the incubation period, splitting the exposed phase into three consecutive stages results in its duration being gamma distributed which is more realistic than the exponential distribution corresponding to a single compartment.

Infections might be reported (*I*) or remain undetected (*U*). The compartment *I* also accounts for severe infections, which might lead to death, the assumption being that all severe cases will be detected. Deceased (*D*) and recovered (*R*) individuals are removed from the chain of transmission, assuming long lasting immunity upon recovery. Susceptible individuals can be infected via contacts with presymptomatic (transmission rate *β*_*E*_), undetected (transmission rate *β*_*U*_), or detected (*β*_*I*_) infectious individuals. We assume that presymptomatic and undetected infectious persons, lacking knowledge about being infectious, do not restrict their contacts to others, and therefore have higher transmission rates than detected infected individuals (*β*_*E*_, *β*_*U*_ > *β*_*I*_) who are expected to quarantine or isolate themselves at least to some degree. Further we include behavioral heterogeneity in the population. We assume that while everyone adheres to moderate restrictions being in place throughout the period under consideration, a certain fraction of the population might not comply with stricter measures as these are applied. Hence, we split the population into two groups, called compliant (subscript *c*) and noncompliant (subscript *n*), respectively. As a simplifying assumption we take the compliant group to perfectly adhere to prescribed contact reductions while the noncompliant group maintains its original contact level, regardless of imposed measures, be it because they do not accept such measures or because they are not able to implement them.

**Fig. 1:**
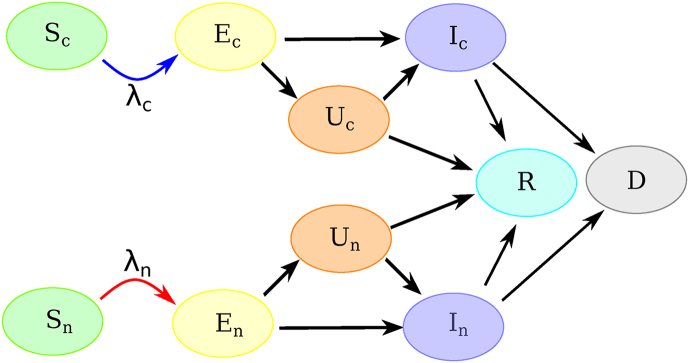
**Model structure for the transmission dynamics of an infectious disease with contact restrictions and partial compliance**. Black arrows indicate transition from one compartment to another, red/blue arrows indicate new infections by virus transmission due to contact with infectious individuals. Upon infection, susceptible (*S*) individuals enter the exposed phase (*E*), divided into three consecutively passed stages, *E*_1_, *E*_2_, *E*_3_, but represented here as a single stage for better clarity. After symptom onset, infections may be detected (*I*) or remain undetected (*U*). Severe cases potentially leading to death are assumed to be always detected. Infected individuals who recovered (*R*) or died (*D*) upon infections, are removed from the chain of transmission. All individuals who are relevant to the disease transmission dynamics are classified as compliant (*S*_*c*_, *E*_*c*_, *U*_*c*_, *I*_*c*_) or noncompliant (*S*_*n*_, *E*_*n*_, *U*_*n*_, *I*_*n*_), depending on their behavior response to imposed contact restrictions.

An overview of the model variables is given in Table 1. The dynamics of the model shown in Fig. 1 is given by the following system of differential equations:

(1) $$\begin{array}{llll} {\dot{S}}_{m} & = & -\lambda_{m}\left(t\right)S_{m} & \\ {\dot{E}}_{1,m} & = & \;\lambda_{m}\left(t\right)S_{m}-\gamma_{E}E_{1,m} & \\ {\dot{E}}_{i,m} & = & \gamma_{E}E_{i-1,m}-\gamma_{E}E_{i,m} & ,i\in \left\{2,3\right\}\\ {\dot{U}}_{m} & = & \left(1-\tau_{m}\right)\gamma_{E}E_{3,m}-\left(\gamma_{U}+\eta_{m}\right)U_{m} & \\ {\dot{I}}_{m} & = & \;\tau_{m}\gamma_{E}E_{3,m}+\eta_{m}U_{m}-\gamma_{I}I_{m} & \\ {\dot{R}}_{m} & = & \left(1-\delta \right)\gamma_{I}I_{m}+\gamma_{U}U_{m} & \\ {\dot{D}}_{m} & = & \;\delta \gamma_{I}I_{m}, & \end{array}$$

for *m*, *k* ∈{*c*, *n*}, and with

(2) $$\lambda_{m}=\underset{k=c,n}{\sum }\left(\beta_{km,E}E_{3,k}+\beta_{km,U}U_{k}+\beta_{km,I}I_{k}\right).$$

**Table 1:** Model variables.

Notation	Description
*S*_*c*/*n*_	compliant/noncompliant susceptible individuals
*E*_*i*,*c*/*n*_	compliant/noncompliant exposed individuals in stage *i* = 1, 2 (not yet contagious)
*E*_3,*c*/*n*_	compliant/noncompliant exposed individuals in stage 3 (already contagious)
*U*_*c*/*n*_	compliant/noncompliant undetected infectious individuals
*R*	recovered individuals
*D*	deceased individuals

The force of infection, *λ*_*m*_, is determined by the specific transmission rates

(3) $$\beta_{km,X}=\beta_{0}\mu_{X}a_{k}s_{m}$$

between the infectious compartment *X* ∈{*E*_3,*k*_, *I*_*k*_, *U*_*k*_∣*k* ∈{*n*, *c*}}, and the susceptible class *S*_*m*_, *m* ∈{*c*, *n*}. Here *β*_0_ denotes a basic transmissibility rate specific to the virus, *a*_*k*_ is the specific infectiousness of population *k* (determined by their social behavior, in particular compliance with restriction rules), *s*_*m*_ the specific susceptibility of the susceptibles *S*_*m*_ (determined by their behavior), and *μ*_*X*_ is the specific weight of infectiousness for stage *X* of the infection. Disease progression through the different infectious stages is given by the rates *γ*_*X*_, that is, 1/*γ*_*X*_ is the average duration of the stage *X*. The incubation time 3/*γ*_*E*_ is split equally among the three compartments *E*_*j*_. Detection may occur with probability *τ*_*m*_ by the end of the incubation period. Later detection, when individuals are already in compartment *U*_*m*_, may depend on the compliant/noncompliant status, *m*, of the infectious person and occurs with rate *η*_*m*_. Detected individuals might die with probability *δ*.

For the simulations shown below we set the total population *N* to approximately 83 million, roughly Germany's population. A list of values for the other model parameters can be found in Appendix D.

### 2.1 The basic reproduction number $R_{0}$ for the pre-intervention phase

As in the pre-intervention phase both the compliant and the noncompliant group are assumed to behave the same, for easiness of notation we omit the compliant/noncompliant index in the following computation. To employ the next generation matrix (NGM) approach for calculation of the basic reproduction number $R_{0}$ (Diekmann et al., 1990), we split the compartments into infected ($x={\left(E_{1},E_{2},E_{3},U,I\right)}^{T}$) and non-infected (*y* = (*S*,*R*,*D*)^*T*^) and write system (1) as

$$\begin{array}{lll} x^{'} & = & F\left(x,y\right)-V\left(x,y\right)\\ y^{'} & = & g\left(x,y\right) \end{array}$$

where F captures the inflow of new individuals into *x* from the non-infected compartments,

$$F\left(x,y\right)={\left(\lambda S,0,0,0,0\right)}^{T},$$

while V collects the progression within the infected compartments as well as the outflow (recovery, deaths). Linearizing the equation for *x* about the disease free equilibrium (DFE) $\overset{-}{x}=0$, $\overset{-}{y}=\left(N,0,0\right)$, we obtain

$$x^{\prime }\approx Fx-Vx$$

where

$$V=\left(\begin{array}{ccccc} \gamma_{E} & 0 & 0 & 0 & 0\\ -\gamma_{E} & \gamma_{E} & 0 & 0 & 0\\ 0 & -\gamma_{E} & \gamma_{E} & 0 & 0\\ 0 & 0 & -\left(1-\tau \right)\gamma_{E} & \gamma_{U}+\eta & 0\\ 0 & 0 & -\tau \gamma_{E} & -\eta & \gamma_{I} \end{array}\right)\;,\;\;\;\;\;\;\text{ and }\;\;\;\;\;\;F=\left(\begin{array}{ccccc} 0 & 0 & \beta_{E} & \beta_{U} & \beta_{I}\\ 0 & 0 & 0 & 0 & 0\\ 0 & 0 & 0 & 0 & 0\\ 0 & 0 & 0 & 0 & 0\\ 0 & 0 & 0 & 0 & 0 \end{array}\right),$$

are the Jacobians of F and V, respectively, evaluated at the DFE. For the DFE to be locally asymptotically stable, all eigenvalues of *F* − *V* must lie in the left half plane, or equivalently, the dominant eigenvalue of *FV*^−1^ must be smaller than 1. The dominant eigenvalue being the first entry of *FV*^−1^, corresponds indeed to the basic reproduction number $R_{0}$ (Diekmann et al., 1990). Short computation leads to

$$R_{0}=R_{I}+R_{U}+R_{E},$$

with

(4) $$R_{I}=\frac{\beta_{I}\left(\left(\eta +\gamma_{U}\right)\tau +\eta \left(1-\tau \right)\right)}{\left(\eta +\gamma_{U}\right)\gamma_{I}}=\frac{\beta_{I}\left(\eta +\tau \gamma_{U}\right)}{\left(\eta +\gamma_{U}\right)\gamma_{I}},\;\;\;\;R_{U}=\frac{\beta_{U}\left(1-\tau \right)}{\eta +\gamma_{U}},\;\;\;\;R_{E}=\frac{\beta_{E}}{\gamma_{E}}.$$

Let us now go back to the distinction into compliant and noncompliant groups. We shall assume that strict control measures are introduced in a very early phase of the outbreak (when we are very close to the DFE) and denote by *ρ* the fraction of individuals that comply with the measures. That is we introduce one infectious individual in an entirely susceptible population split into compliant, *S*_*c*_(0) = *ρN*, and noncompliant, *S*_*n*_(0) = (1 − *ρ*)*N*, group. Compliance corresponds to a reduction of effective contacts to a fraction *r* ∈ [0, 1] of the original value. With these notations the initial controlled reproduction number would be

(5) $$R_{c}=\left(1-\left(1-r\right)\rho \right)R_{0}.$$

The straightforward but somewhat lengthy derivation is given in the appendix. In Fig. 6b we show the ratio between $R_{c}$ and $R_{0}$ in dependence of *ρ* and *r*. The trivial limit cases are (i) *r* = 0 and *ρ* = 1, that is full compliance and reduction to zero contacts, yielding $R_{c}=0$, and (ii) *r* = 1, that is no intervention, or *ρ* = 0, no compliance, yielding $R_{c}=R_{0}$.

## 3 Results

The simulations that we show below are not calibrated on any specific time series but parametrized in a way to approximately reproduce the COVID-19 dynamics in different phases of the pandemic. We start the simulations with initial low incidence under moderate control measures, such that the resulting reproduction number is slightly larger than one. For a certain initial period, both the compliant and the noncompliant group behave the same, that is, the two subpopulations have the same transmission rates (*β*_*km*,*J*_ = *β*_*lp*,*J*_, for all *k*, *l*, *m*, *p* ∈{*c*, *n*}, *J* ∈{*E*, *U*, *I*}). After this initial phase we assume that transmission rates, hence the reproduction number, slightly increase over time. In the context of COVID-19, this setting might mimic the transition from the controlled situation in the summer 2020 to the fall 2020 in Germany and other European countries. We suppose that stricter intervention measures aiming at the reduction of transmission are introduced when a daily incidence of ≈ 20, 000 cases is reached. As an effect of these control measures, contacts in the population should significantly decrease and, if the whole population was behaving in compliance with the prescribed measures, contact rates would be reduced to a certain fraction *r* < 1 of their value before intervention. Upon the introduction of stricter control measures the compliant and noncompliant groups start behaving differently: noncompliants maintain their behavior (activity and susceptibility). In other words, if the whole population was noncompliant, the reproduction number would remain the same as before intervention. By a slight abuse of notation we shall denote by $R_{0}$ the reproduction number before the modeled intervention. Here we set $R_{0}\approx 1.5$, approximately the value estimated for COVID-19 in late summer/early fall 2020 in Germany (Robert Koch Institute Coronavirus Disease 2019 (COVID-19), 2019). This value being already affected by some control measures, it is indeed significantly smaller than the uncontrolled reproduction number of SARS-CoV-2, mostly estimated above 2 (Kucharski et al., 2020; Zhao et al., 2020).

### 3.1 Scenarios for different contact reduction and compliance levels

In the following we show different scenarios for the dynamics of the outbreak under the variation of two major unknown factors:

1. **Reduction of contacts.** We assume that in accordance with control measures contacts would be reduced *by* a factor 75%, 50%, or 20% of the level previous interventions. This would lead, in case of perfect compliance, to a reduction *to* 25%, 50%, or 80% of the reproduction number $R_{0}$ before interventions, corresponding to *r* = 0.25, 0.5, or 0.8, respectively.
2. **Compliant fraction of the population.** We vary the fraction *ρ* ∈ [0, 1] of the population complying with restriction measures. We assume that the differentiation into compliant/noncompliant individuals occurs only once, namely at the time of intervention, and that individuals do not switch to the opposite behavior (noncompliant/compliant) for the entire course of the simulations.

It is yet unclear how many secondary cases of COVID-19 result from presymptomatic transmission, with estimates ranging from 6.4% (Wei et al., 2020) to 46*%* (Ferretti et al., 2020, He et al., 2020), or even above 50*%* (Ganyani et al., 2020). It is however widely accepted that almost half the infections are transmitted from pre- or asymptomatic infectious individuals (Buitrago-Garcia et al., 2020; Johansson et al., 2021). If we further assume that detected cases are well isolated and significantly reduce their contacts, these individuals will contribute only marginally to disease transmission. We therefore fix the relative infectiousness parameters (*μ*_*X*_, *X* ∈{*E*, *U*, *I*}) in the definition of *β*_*km*,*X*_ such that

$$R_{U}⪆R_{E}\gg R_{I},$$

that is, individuals in *U* contribute slightly more to $R_{0}$ than those in *E*_3_, and the contribution of individuals in *I* is dwarfed by both of them. Given the other parameters listed in Appendix D, we achieve (i) $R_{I}$ barely contributing by choosing *μ*_*I*_ = 0.1*μ*_*U*_ and (ii) $R_{E}$ accounting for a little less than 40*%* of $R_{0}$ by fixing *μ*_*E*_ = 1.5*μ*_*U*_.

For the following scenarios we furthermore consider two possible assumptions for the time of detection. We show as next the *late detection setting*, with *τ* = 0.1 (corresponding to 10*%* of cases detected by symptom onset) and *η* = 0.6*γ*_*U*_ (≈38*%* of *U* detected) at low prevalence. This leads to approximately 45*%* detection as long as the prevalence is sufficiently small. We further assume that the detection rate decreases in the face of large numbers of (undetected) cases due to finite testing capacity, e.g.,

(6) $$\eta =\overset{-}{\eta }\frac{K}{U_{c}+U_{n}+\alpha E_{3,c}+\alpha E_{3,n}+K}$$

for some constant *K* and a maximal detection rate $\overset{-}{\eta }$. As long as the total number of undetected infectious individuals is small as compared to *K*, the correction factor is close to one, $\eta \approx \overset{-}{\eta }$, but as the prevalence approaches the order of *K*, an increasing proportion of infections goes undetected. A similar discussion applies to *τ* which is similarly composed of $\overset{-}{\tau }=0.1$ and a similar correction factor as derived for *η*. We remark that this nonlinearity does not affect the calculation of $R_{0}$ or $R_{c}$ but may well affect the reproduction number *R*_*t*_ far away from the disease free equilibrium. This makes intuitive sense: Since detected infectious individuals are assumed to (self-) isolate and produce few secondary infections after being detected, any reduction in detection rates will accelerate the spread of the epidemic while at the same time making reported case numbers appear smaller. A motivation for the precise shape of the correction factor is given in the appendix. An *early detection setting*, with *τ* = 0.3 (corresponding to 30*%* of cases detected by symptom onset), $\overset{-}{\eta }=0.25\gamma_{U}$ (corresponding to 20*%* of *U* detected), yielding a comparable total detection ratio, was also considered, but simulations are shown or discussed only when differences with the late detection setting are significant.

#### 3.1.1 Scenario 1: $R_{c}=0.25R_{0}$ at full compliance

**Fig. 2:**
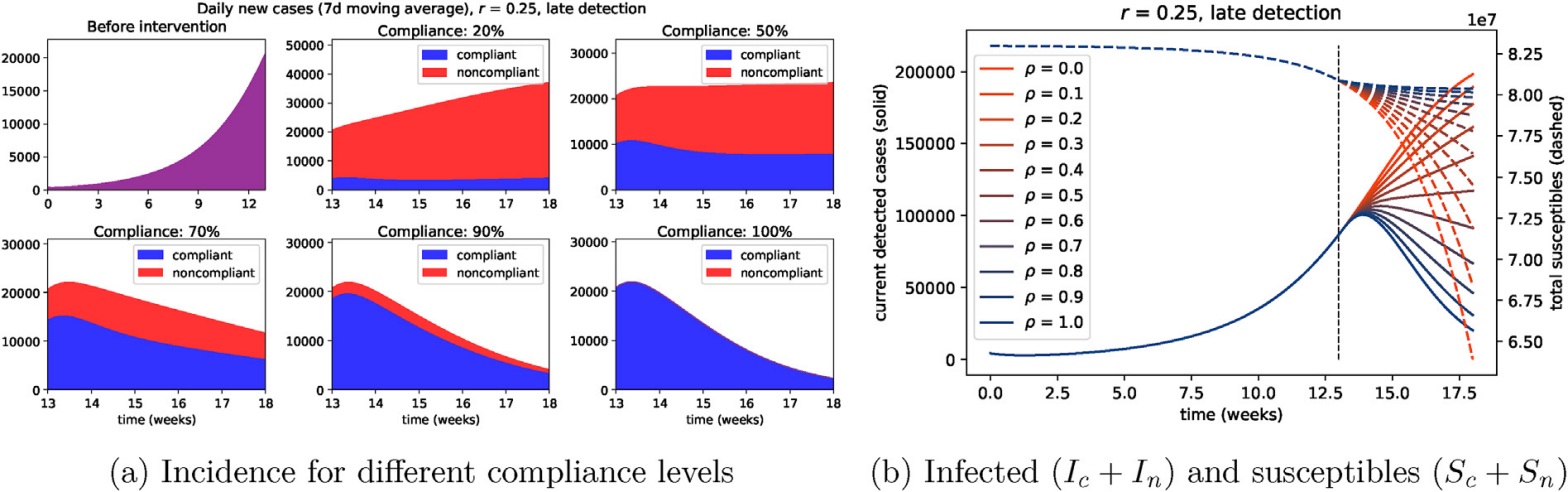
**Scenario 1:**$R_{c}=0.25R_{0}$. The upper left panel in (a) shows the incidence before the intervention. For different compliant fractions (*ρ*) of the total population we show (a) the evolution in time of daily cases reported among compliant (blue) and noncompliant (red) individuals, and (b) the currently known active cases (*I*_*c*_ + *I*_*n*_, continuous curves) and susceptible individuals (*S*_*c*_ + *S*_*n*_, dashed curves). The vertical dashed line shows the time of intervention. The effect of contact reduction is not immediately evident since new infections are not detected until several days later. Notice that the daily new cases in (a) are shown as 7-day moving average, meaning that weekly oscillations due to lower reporting rates on weekends are smoothed over and do not show in the plots.

The first scenario considered is a prescribed contact reduction that would lead to a control reproduction number $R_{c}$ being at 25*%* of $R_{0}$. Assuming an initial $R_{0}\approx 1.5$, this would lead to $R_{c}\approx 0.375$. The results are shown in Fig. 2. We observe that at full compliance (100*%* of the population, *ρ* = 1), the incidence would indeed quickly decrease. For lower levels of compliance, the decrease is expectedly slower. However, if only half the population adheres to the measures (50*%* compliance), this contact reduction is only sufficient to stall the rising incidence. This happens because the *effective reproduction number* *R*_*t*_, resulting from transmission rates of compliant and noncompliant populations under the implemented control measures, approaches values close to 1 about two weeks after intervention. Fig. 2a evidences that the 50*%* noncompliant individuals make up way more than 50*%* of the cases. At even lower compliance, the measures may help to slow down the increase but are no longer sufficient to stop it.

In Fig. 2b, the red curve (*ρ* = 0) indicates the course of the epidemic with no compliance at all with the NPIs introduced in week 13. This corresponds to a situation without any new intervention measures, and five weeks after the (non-)intervention, already some 25*%* of the population would have been infected (cf. the dashed curve for the susceptible population). The number of detected cases does not rise as quickly as the loss of susceptibles would suggest. This is due to the limited test capacity, cf. (6), and the decreasing detection ratio as the prevalence becomes too large.

#### 3.1.2 Scenario 2: $R_{c}=0.5R_{0}$ at full compliance

**Fig. 3:**
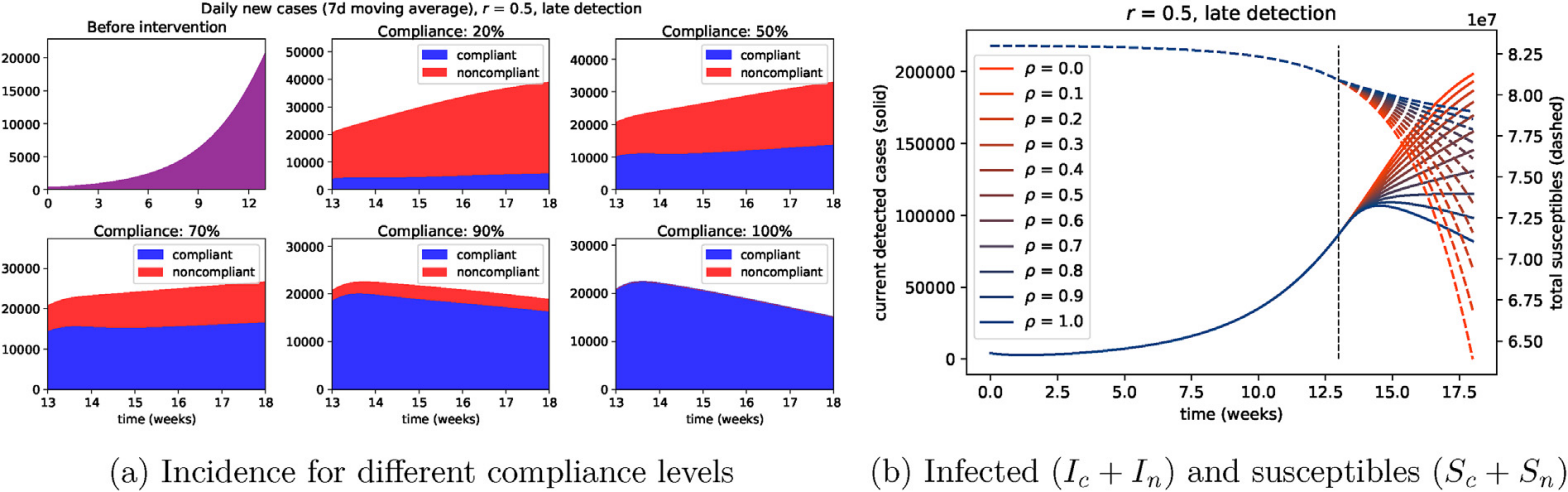
**Scenario 2:**$R_{c}=0.5R_{0}$. The upper left panel in (a) shows the incidence before the intervention. For different compliant fractions (*ρ*) of the total population we show (a) the evolution in time of daily cases reported among compliant (blue) and noncompliant (red) individuals, and (b) the currently known active cases (*I*_*c*_ + *I*_*n*_, continuous curves) and susceptible individuals (*S*_*c*_ + *S*_*n*_, dashed curves). The vertical dashed line shows the time of intervention. The effect of contact reduction is not immediately evident since new infections are not detected until several days later.

The second scenario, shown in Fig. 3, assumes that at 100*%* compliance, the transmission rates and hence the control reproduction number would be cut in half, leading to $R_{c}\approx 0.75$ at $R_{0}=1.5$. Still, at perfect compliance, the incidence would start falling several days after the intervention but now even 70*%* compliance would not be sufficient to prevent the case numbers from rising.

#### 3.1.3 Scenario 3: $R_{c}=0.8R_{0}$ at full compliance

**Fig. 4:**
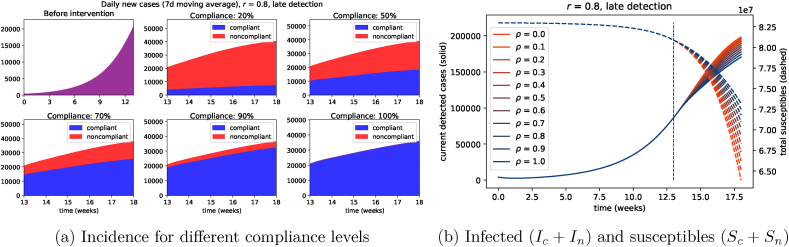
**Scenario 3:**$R_{c}=0.8R_{0}$. The upper left panel in (a) shows the incidence before the intervention. For different compliant fractions (*ρ*) of the total population we show (a) the evolution in time of daily cases reported among compliant (blue) and noncompliant (red) individuals, and (b) the currently known active cases (*I*_*c*_ + *I*_*n*_, continuous curves) and susceptible individuals (*S*_*c*_ + *S*_*n*_, dashed curves). The vertical dashed line shows the time of intervention. The limited effect of contact reduction is not stopping the increase in daily new cases and there is no qualitative difference between full compliance (*ρ* = 1) and full noncompliance (*ρ* = 0).

The implementation of moderate measures, reducing the transmission rate among compliant individuals by only 20*%*, leads to a reproduction number of $R_{c}\approx 1.2$ at perfect compliance. As shown in Fig. 4, this is not sufficient to stop the increasing case numbers even if the whole population would adhere to the measures. The effect is due to the limited efficacy of the control measures (reducing the reproduction number from 1.5 to at best 1.2), rather than to the level of compliance.

#### 3.1.4 Scenario 2′: $R_{c}=0.5R_{0}$ at full compliance, with early detection

**Fig. 5:**
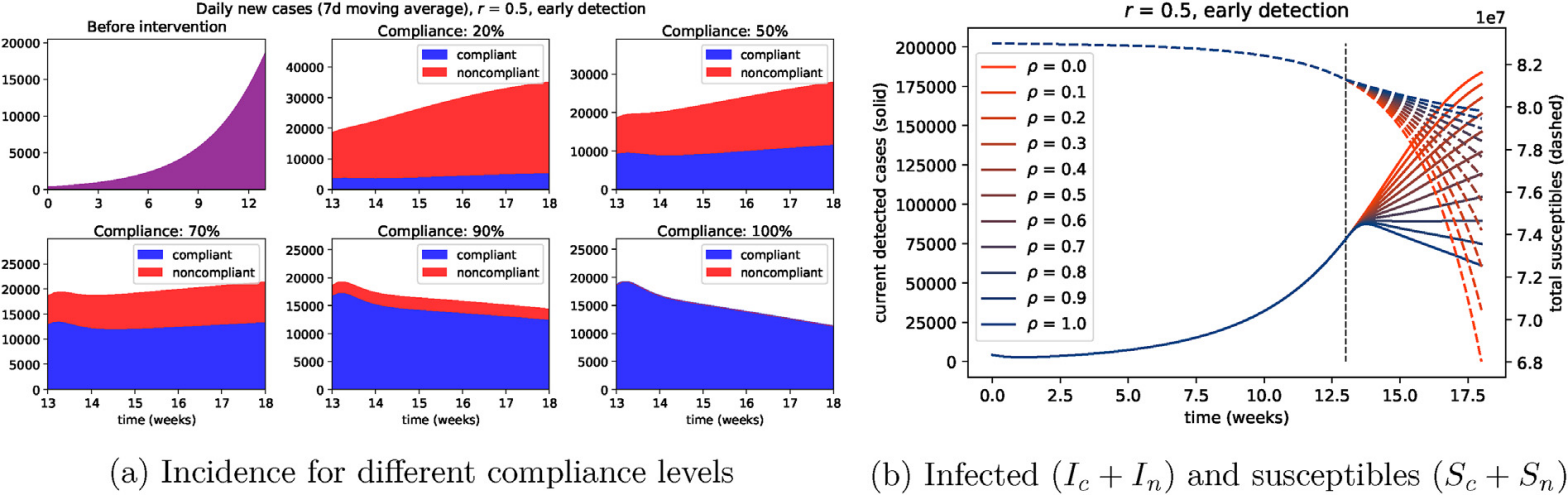
**Scenario2′:**$R_{c}=0.5R_{0}$. If infections are detected in an early stage, the case incidence follows closely the true incidence of new infections. The upper left panel in (a) shows the incidence before the intervention. For different compliant fractions (*ρ*) of the total population we show (a) the evolution in time of daily cases reported among compliant (blue) and noncompliant (red) individuals, and (b) the currently known active cases (*I*_*c*_ + *I*_*n*_, continuous curves) and susceptible individuals (*S*_*c*_ + *S*_*n*_, dashed curves). The vertical dashed line shows the time of intervention. Notice (panel (a)) the drop in incident cases at 70*%* compliance, followed by a slow increase. The reasons for this rebound will be discussed in Appendix C. Due to the shorter detection delay, the number of active detected cases drops (in case of high compliance) faster than in the corresponding settings in Fig. 3a.

The assumptions on the reproduction numbers are the same as in Scenario 2, but here we consider the case of earlier detection (*τ* = 0.3, *η* = 0.25*γ*_*U*_). Qualitatively, the results shown in Fig. 5 are the same as in Scenario 2 (cf. Fig. 3), that is, for compliance levels below 50*%* the daily new cases keep increasing. Due to early case detection, however, the incidence of detected cases follows more closely the time course of “actual” new daily infections (*γ*_*E*_(*E*_3,*c*_ + *E*_3,*u*_)). A consequence of this effect is that for 50*%* compliance, for which *R*_*t*_ is rather close to 1, after an initial drop the incidence increases again after a few days. The initial brief drop reflects transiently falling new infections. In case of late detection (cf. Fig. 2a, compliance 50*%*) this effect is smoothed out and not visible in the daily incidence time series.

**Fig. 6:**
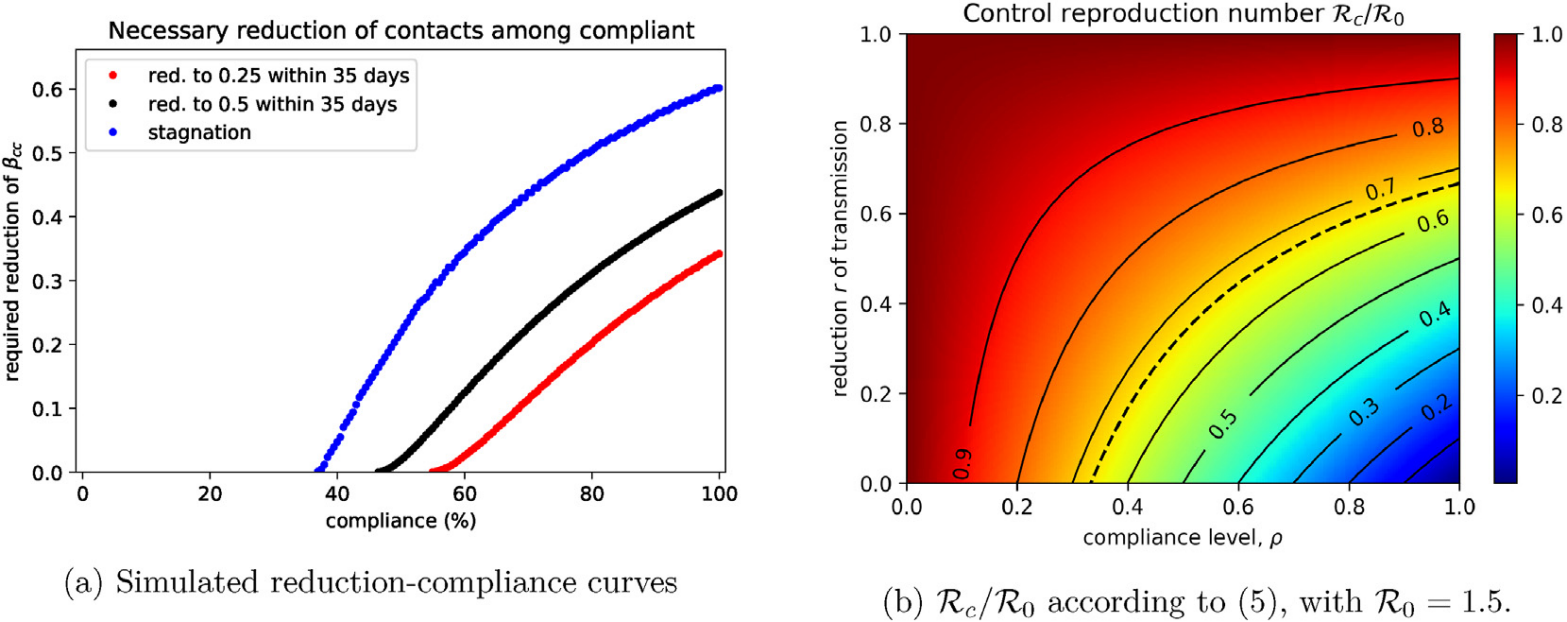
(a) Required reduction of transmission among compliant individuals in order to achieve stagnation (blue) or a desired reduction within five weeks to 25*%* (red) or 50*%* (black) of case incidence compared to the value at the time of intervention. (b) Ratio between the control reproduction number, $R_{c}$, and the pre-intervention reproduction number, $R_{0}$, according to (5). Notice that reaching $R_{c}=1$ from $R_{0}=1.5$ implies a ratio of 2/3 and that the corresponding level set (dashed line) is indeed very similar to the curve of required reductions for achieving stagnating incidence in (a). This level set being shifted to the left reflects the fact that the simulations do not operate at the DFE.

### 3.2 Reduction levels necessary to either significantly reduce incidence or afford stagnation

So far, we have seen how the incidence of new detected cases progresses at different compliance levels if a given reduction of effective contacts among compliant individuals is prescribed. Let us now take a different point of view. For a given compliance level we ask how strong the reduction in transmission among compliant individuals needs to be in order to

(*i*) reduce the incidence to 25*%* or 50*%* of the value at intervention time within 5 weeks after intervention, or
(*ii*) reach permanent stagnation of incidence, meaning that after 3 weeks post intervention the incidence shall not increase anymore beyond a small tolerance of 0.25*%* over the last 10 days of the simulated period.

To this end, we performed simulations for 201 compliance levels (0*%* through 100*%* in steps of 0.5 percentage points) and screened for the reduction levels sufficient to achieve either of the goals (*i*) or (*ii*). Results are shown in Fig. 6. Since we start with a reproduction number $R_{0}$ (pre-intervention) of about 1.5, hence rising incidence, it is not surprising that for low compliance even the complete elimination of contacts among compliant individuals (*β*_*cc*_ ≈ 0) is not sufficient to achieve either one of the above goals. The population of susceptible noncompliant individuals is still sufficiently large and a significant reduction in the daily incidence is not feasible. At compliance levels close to 40*%* a very strong reduction in transmission among compliant individuals (*β*_*cc*_ ≈ 0) allows to stall the rising incidence. In order to achieve the desired 50*%* reduction in daily cases, as stated in point (*i*), a compliance level of nearly 50*%* or more is necessary. Reducing the incidence to 25*%* within the same time requires even higher compliance levels. Conversely, even at full compliance, the contact reductions need to be sufficiently large in order to achieve stagnating or falling incidence. This is in agreement with what was already shown in Fig. 4, where a reduction of effective contacts by 20*%* was not sufficient to keep the incidence from rising further. Here, we see that reduction of effective contacts by a factor 0.4 to 0.6 of the pre-intervention level is required to either reach stagnation or significant reduction of daily incidence. Qualitatively, the same conclusions are also obvious from consulting the reproduction numbers shown for comparison in Fig. 6b.

**Fig. A1:**
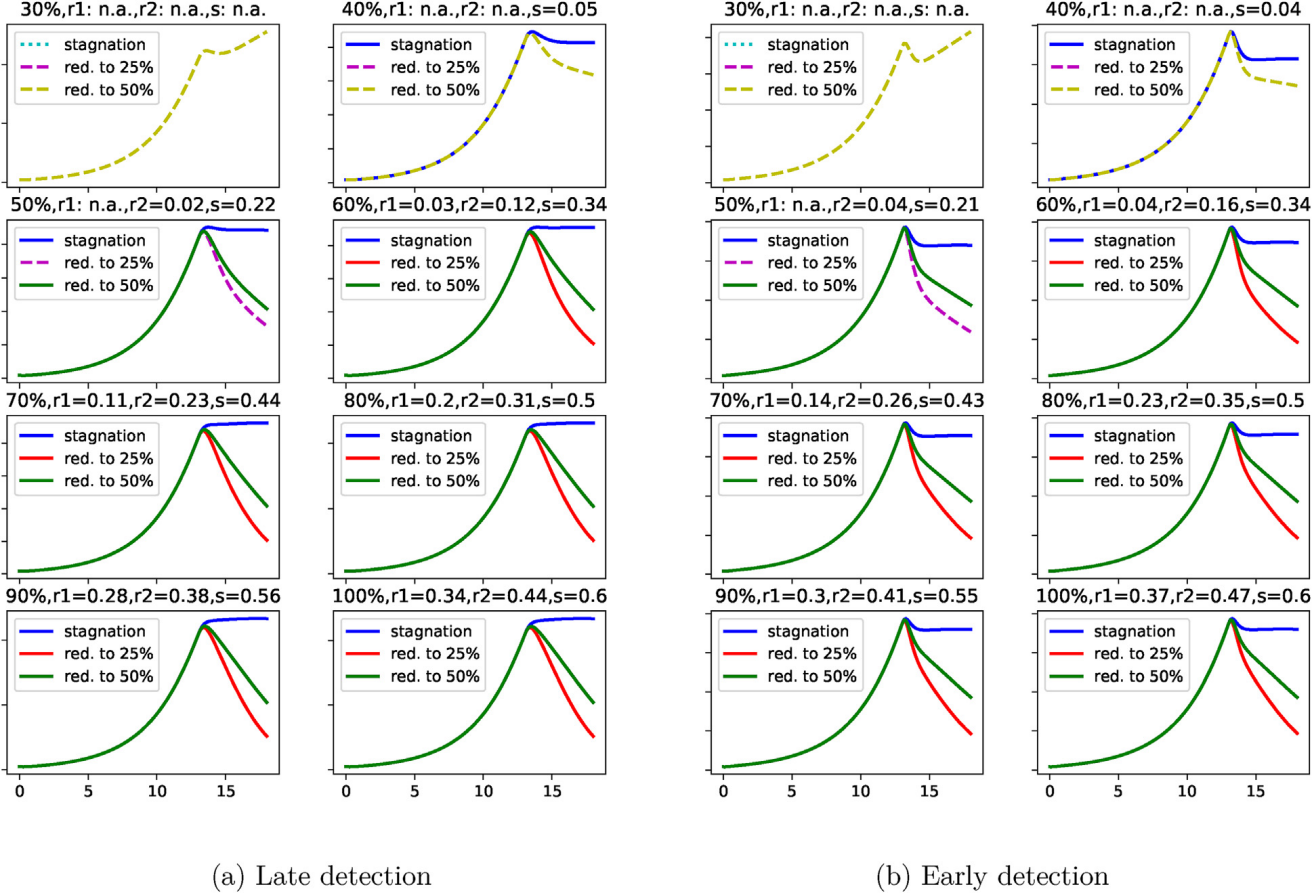
Daily incidence for threshold cases in Fig. 6a. For different compliance levels (indicated as percentage on the top of each panel), the incidence over time is shown for the critical reductions according to Fig. 6a. The values *r*1 and *r*2 indicate the reduction of the transmission rate among compliant individuals required for lowering the incidence to 25*%* or 50*%*, respectively, of the value at the time of intervention, whereas *s* is the reduction factor required for stagnation as explained in Subsection 3.2. If a certain goal cannot be attained (denoted by “n.a.“) at the given compliance level, the incidence for the *r* = 0, meaning that compliant individuals do not participate in the transmission at all, is shown as a dashed line.

Simulated curves for the evolution of daily incidence in time corresponding to the limit cases (stagnation or reduction to a fixed percentage of pre-intervention value just being achieved) in Fig. 6a are shown in the appendix, Fig. A1a.

## 4 Conclusion and discussion

In order to illustrate possible effects of less than perfect compliance with non-pharmaceutical interventions (NPIs) on their effectiveness in curbing the spread of infectious diseases, we modeled and simulated a situation mimicking the status of the COVID-19 epidemic in Germany in the fall of 2020. The model captures both (i) reduced susceptibility of individuals adhering to the proposed NPIs (an effect similar to protection which could be achieved via vaccination) and (ii) reduced transmission from compliant individuals (acting similar to quarantine or treatment, cf. Chapter 9 in (Martcheva, 2015)). The simulations show that in implementing NPIs to rapidly reduce daily cases, a concurrence of a sufficient level of compliance (*ρ*) in the population and a significant reduction, *r*, of effective contacts among compliant individuals is required. For example, let the pre-intervention reproduction number be about 1.5, and let both the effective infectiousness, *a*_*c*_, and the susceptibility, *s*_*c*_, of half the population (compliance level *ρ* = 0.5) be reduced by about 30*%* each. This leads to a 51*%* reduction of transmission among compliant individuals, cf. (3), *r* = 0.49, which is by far not enough to stop the rise in new cases, as can be seen in the upper right panel in Fig. 3a. Only if the measures are sufficiently effective in reducing transmission *and* a large proportion of the population implements such measures, a stagnation or even reduction of case numbers can be achieved in a reasonably short time (in the above simulations: 5 weeks).

Moreover, if infections are detected and reported rather quickly (Scenario 2′, Fig. 5), reduced transmission among compliant individuals can lead to a brief reduction in newly reported cases before these start rising again (cf. Fig. 5 compliance 50% or 70%). If reported cases are used as a daily proxy for evaluating the effectiveness of control measures, such a short-time decline could be misleading.

The system (1) for transmission dynamics used for the above simulations was developed as a simplification of our previous models for COVID-19 in Germany (Barbarossa et al., 2020; Barbarossa & Fuhrmann, 2021). For the sake of simplicity we decided not to include age groups, stages of infection, hospitalizations or cases requiring intensive care, nor considered any reduction of contact rates due to self-control of individuals to high incidence values (Capasso & Serio, 1978; Fenichel et al., 2011). Further we assume that individuals are either compliant or noncompliant for the whole duration of control measures, and that there is no behavioral switching between the two groups. The model could be extended to include such a switch, as was done in the past by other authors (Poletti et al., 2009; D'Onofrio & Manfredi, 2020; Iboi et al., 2021).

We should note that all the simulations discussed above presume that most of the population is still susceptible at the time of intervention. This leads to the effective reproduction number *R*_*t*_ being only slightly smaller than the basic reproduction number $R_{0}$ (before intervention) or the control reproduction number $R_{c}$ (after intervention). This is one reason why the theoretical threshold curve shown in Fig. 6b is rather close to those found in Fig. 6a showing the required reduction of transmission among compliant individuals for given compliance levels. Clearly, the smaller the susceptible fraction of the population is at the beginning, the faster the relative change in the number of susceptibles over time and the more pronounced will the effect of depleting the pool of susceptibles be.

## Declaration of competing interest

The authors declare that they have no known competing financial interests or personal relationships that could have appeared to influence the work reported in this paper.

## Figures and Tables

**Fig. A2:**
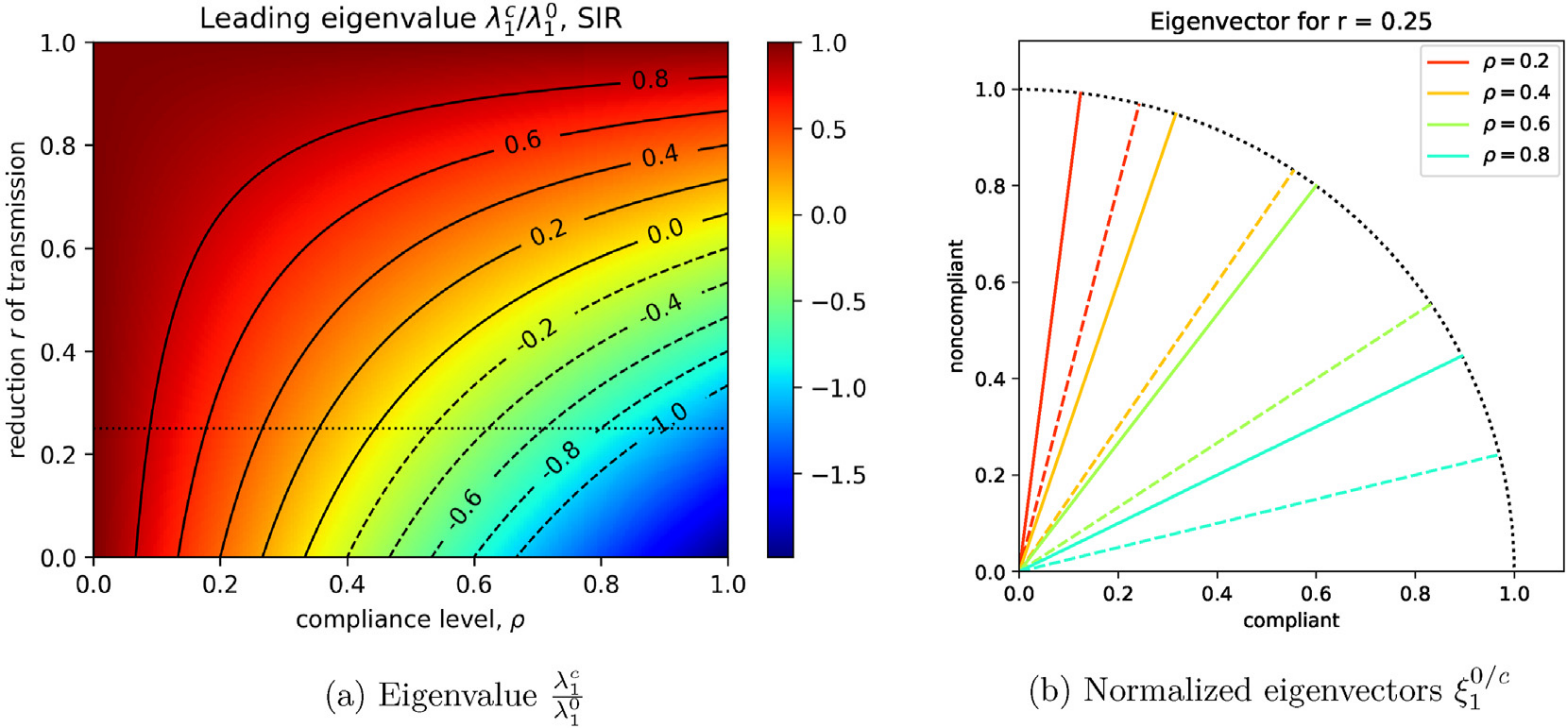
Illustration of the leading eigenvalue $\lambda_{1}^{c}$ (left) of *J* as multiple of $\lambda_{1}^{0}$, depending on the transmission reduction factor, *r*, and the compliance level, *ρ*. Notice that the level $\lambda_{1}^{c}=0$ corresponds to $R_{c}=1$ for system (A1). On the right, the corresponding eigenvector, $\xi_{1}^{c}$, in the *I*_*c*_-*I*_*n*_ plane is shown for *r* = 0.25 and $a_{c}=s_{c}=\sqrt{r}=0.5$, corresponding to dotted line in (a), and different compliance levels. For comparison, the dashed lines depict the eigenvectors $\xi_{1}^{0}$ at the same compliance levels. It is obvious that the eigenvectors $\xi_{1}^{c}$ are shifted towards *I*_*n*_. Notice that *ρ* = 0.4 approximately corresponds to $\lambda_{1}^{c}=0$ at *r* = 0.25. The colors of the eigenvectors correspond to those of the point in (a) where the pair (*ρ*, *r* = 0.25) is located.

**Fig. A3:**
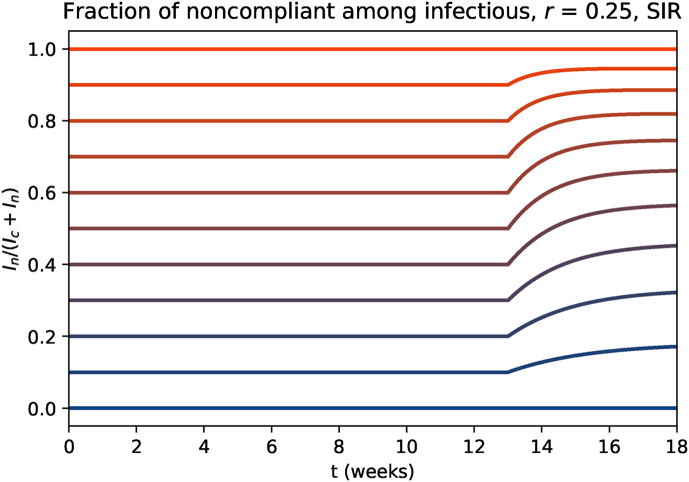
Before the intervention (week 13), the fraction of noncompliant among all currently infectious individuals is precisely the same as their fraction among the whole population. After intervention, the share of noncompliant infectious individuals rises in accordance with the shifted eigenvector illustrated in Fig. A2b.

## Appendix A Calculation of the control reproduction number

For the calculation of the control reproduction number $R_{c}$ we apply the next generation matrix (NGM) approach from (Diekmann et al., 1990). The population can be split into not infected compartments, $y={\left(S_{c},S_{n},R,D\right)}^{T}$, and infected compartments $x={\left(E_{1,c},E_{1,n},E_{2,c},E_{2,n},E_{3,c},E_{3,n},U_{c},U_{n},I_{c},I_{n}\right)}^{T}$. The disease free equilibrium is

$$y={\left(\rho N,\left(1-\rho \right)N,0,0\right)}^{T},\;\;\;\;\;\;\;x=0$$

where *ρ* denotes the compliance level, i.e., the fraction of the population that is compliant. By the NGM approach we construct the 10 × 10 matrices

$$F=\beta_{0}\left(\begin{array}{cccccccccc} 0 & 0 & 0 & 0 & \mu_{E}a_{c}s_{c}\rho & \mu_{E}a_{n}s_{c}\rho & a_{c}s_{c}\rho & a_{n}s_{c}\rho & \mu_{I}a_{c}s_{c}\rho & \mu_{I}a_{n}s_{c}\rho \\ 0 & 0 & 0 & 0 & \mu_{E}a_{c}s_{n}\hat{\rho } & \mu_{E}a_{n}s_{n}\hat{\rho } & a_{c}s_{n}\hat{\rho } & a_{n}s_{n}\hat{\rho } & \mu_{I}a_{c}s_{n}\hat{\rho } & \mu_{I}a_{n}s_{n}\hat{\rho }\\ 0 & 0 & 0 & 0 & 0 & 0 & 0 & 0 & 0 & 0\\ 0 & 0 & 0 & 0 & 0 & 0 & 0 & 0 & 0 & 0\\ 0 & 0 & 0 & 0 & 0 & 0 & 0 & 0 & 0 & 0\\ 0 & 0 & 0 & 0 & 0 & 0 & 0 & 0 & 0 & 0\\ 0 & 0 & 0 & 0 & 0 & 0 & 0 & 0 & 0 & 0\\ 0 & 0 & 0 & 0 & 0 & 0 & 0 & 0 & 0 & 0\\ 0 & 0 & 0 & 0 & 0 & 0 & 0 & 0 & 0 & 0\\ 0 & 0 & 0 & 0 & 0 & 0 & 0 & 0 & 0 & 0 \end{array}\right)$$

where we wrote the transmission rates as *β*_*mk*,*X*_ = *β*_0_*μ*_*X*_*a*_*m*_*s*_*k*_ and abbreviated $\hat{\rho }=\left(1-\rho \right)$, and

$$V\;=\;\left(\begin{array}{cccccccccc} \gamma_{E} & 0 & 0 & 0 & 0 & 0 & 0 & 0 & 0 & 0\\ 0 & \gamma_{E} & 0 & 0 & 0 & 0 & 0 & 0 & 0 & 0\\ -\gamma_{E} & 0 & \gamma_{E} & 0 & 0 & 0 & 0 & 0 & 0 & 0\\ 0 & -\gamma_{E} & 0 & \gamma_{E} & 0 & 0 & 0 & 0 & 0 & 0\\ 0 & 0 & -\gamma_{E} & 0 & \gamma_{E} & 0 & 0 & 0 & 0 & 0\\ 0 & 0 & 0 & -\gamma_{E} & 0 & \gamma_{E} & 0 & 0 & 0 & 0\\ 0 & 0 & 0 & 0 & -\left(1-\tau_{c}\right)\gamma_{E} & 0 & \gamma_{U}+\eta_{c} & 0 & 0 & 0\\ 0 & 0 & 0 & 0 & 0 & -\left(1-\tau_{n}\right)\gamma_{E} & 0 & \gamma_{U}+\eta_{n} & 0 & 0\\ 0 & 0 & 0 & 0 & -\tau_{c}\gamma_{E} & 0 & -\eta_{c} & 0 & \gamma_{I} & 0\\ 0 & 0 & 0 & 0 & 0 & -\tau_{n}\gamma_{E} & 0 & -\eta_{n} & 0 & \gamma_{I} \end{array}\right).$$

In what follows, we assume *τ*_*c*_ = *τ*_*n*_ = : *τ* and *η*_*c*_ = *η*_*n*_ = : *η*, meaning that the chance of detection is independent of the behavior.^1^ Then, the inverse of *V* is

$$V^{-1}=\;\left(\begin{array}{cccccccccc} \frac{1}{\gamma_{E}} & 0 & 0 & 0 & 0 & 0 & 0 & 0 & 0 & 0\\ 0 & \frac{1}{\gamma_{E}} & 0 & 0 & 0 & 0 & 0 & 0 & 0 & 0\\ \frac{1}{\gamma_{E}} & 0 & \frac{1}{\gamma_{E}} & 0 & 0 & 0 & 0 & 0 & 0 & 0\\ 0 & \frac{1}{\gamma_{E}} & 0 & \frac{1}{\gamma_{E}} & 0 & 0 & 0 & 0 & 0 & 0\\ \frac{1}{\gamma_{E}} & 0 & \frac{1}{\gamma_{E}} & 0 & \frac{1}{\gamma_{E}} & 0 & 0 & 0 & 0 & 0\\ 0 & \frac{1}{\gamma_{E}} & 0 & \frac{1}{\gamma_{E}} & 0 & \frac{1}{\gamma_{E}} & 0 & 0 & 0 & 0\\ \frac{1-\tau }{\gamma_{U}+\eta } & 0 & \frac{1-\tau }{\gamma_{U}+\eta } & 0 & \frac{1-\tau }{\gamma_{U}+\eta } & 0 & \frac{1}{\gamma_{U}+\eta } & 0 & 0 & 0\\ 0 & \frac{1-\tau }{\gamma_{U}+\eta } & 0 & \frac{1-\tau }{\gamma_{U}+\eta } & 0 & \frac{1-\tau }{\gamma_{U}+\eta } & 0 & \frac{1}{\gamma_{U}+\eta } & 0 & 0\\ \frac{\tau \gamma_{U}+\eta }{\left(\gamma_{U}+\eta \right)\gamma_{I}} & 0 & \frac{\tau \gamma_{U}+\eta }{\left(\gamma_{U}+\eta \right)\gamma_{I}} & 0 & \frac{\tau \gamma_{U}+\eta }{\left(\gamma_{U}+\eta \right)\gamma_{I}} & 0 & \frac{\eta }{\left(\gamma_{U}+\eta \right)\gamma_{I}} & 0 & \frac{1}{\gamma_{I}} & 0\\ 0 & \frac{\tau \gamma_{U}+\eta }{\left(\gamma_{U}+\eta \right)\gamma_{I}} & 0 & \frac{\tau \gamma_{U}+\eta }{\left(\gamma_{U}+\eta \right)\gamma_{I}} & 0 & \frac{\tau \gamma_{U}+\eta }{\left(\gamma_{U}+\eta \right)\gamma_{I}} & 0 & \frac{\eta }{\left(\gamma_{U}+\eta \right)\gamma_{I}} & 0 & \frac{1}{\gamma_{I}} \end{array}\right)\;.$$

The next generation matrix is hence

$$\begin{array}{lll} FV^{-1} & = & \beta_{0}\left(\begin{array}{ccc} \left(\frac{\mu_{I}\left(\tau \gamma_{U}+\eta \right)}{\left(\gamma_{U}+\eta \right)\gamma_{I}}+\frac{1-\tau }{\gamma_{U}+\eta }+\frac{\mu_{E}}{\gamma_{E}}\right)a_{c}s_{c}\rho & \left(\frac{\mu_{I}\left(\tau \gamma_{U}+\eta \right)}{\left(\gamma_{U}+\eta \right)\gamma_{I}}+\frac{1-\tau }{\gamma_{U}+\eta }+\frac{\mu_{E}}{\gamma_{E}}\right)a_{n}s_{c}\rho & *\ldots *\\ \left(\frac{\mu_{I}\left(\tau \gamma_{U}+\eta \right)}{\left(\gamma_{U}+\eta \right)\gamma_{I}}+\frac{1-\tau }{\gamma_{U}+\eta }+\frac{\mu_{E}}{\gamma_{E}}\right)a_{c}s_{n}\left(1-\rho \right) & \left(\frac{\mu_{I}\left(\tau \gamma_{U}+\eta \right)}{\left(\gamma_{U}+\eta \right)\gamma_{I}}+\frac{1-\tau }{\gamma_{U}+\eta }+\frac{\mu_{E}}{\gamma_{E}}\right)a_{n}s_{n}\left(1-\rho \right) & *\ldots *\\ 0 & 0 & 0\ldots 0\\ \vdots & \vdots & \vdots \end{array}\right)\\ & = & \underset{R_{0}}{\underset{︸}{\beta_{0}\left(\frac{\mu_{I}\left(\tau \gamma_{U}+\eta \right)}{\left(\gamma_{U}+\eta \right)\gamma_{I}}+\frac{1-\tau }{\gamma_{U}+\eta }+\frac{\mu_{E}}{\gamma_{E}}\right)}}\left(\begin{array}{ccc} a_{c}s_{c}\rho & a_{n}s_{c}\rho & *\ldots *\\ a_{c}s_{n}\left(1-\rho \right) & a_{n}s_{n}\left(1-\rho \right) & *\ldots *\\ 0 & 0 & 0\ldots 0\\ \vdots & \vdots & \vdots \end{array}\right). \end{array}$$

That the common factor is $R_{0}$ can be noted by recalling that *β*_*X*_ = *μ*_*X*_*β*_0_*as* in the pre-intervention setting. Since we are interested in the dominant eigenvalue of this matrix and all but the first two rows are filled with zeros, we only need to calculate the eigenvalues of the upper 2 × 2 minor

$$\left(\begin{array}{cc} a_{c}s_{c}\rho & a_{n}s_{c}\rho \\ a_{c}s_{n}\left(1-\rho \right) & a_{n}s_{n}\left(1-\rho \right) \end{array}\right).$$

This leads to

$$R_{c}=R_{0}\left(a_{c}s_{c}\rho +a_{n}s_{n}\left(1-\rho \right)\right),$$

which is remarkably independent of the detection parameters, meaning that the threshold for achieving $R_{c}=1$ given any $R_{0}>1$ should be the same for early and late detection, respectively. The ratio between $R_{c}$ and $R_{0}$ in dependence of the level of compliance *ρ* and the fraction *r* = *a*_*c*_*s*_*c*_ to which transmission rates among compliant individuals are reduced after intervention is shown in Fig. 6b.

Notice that this approach allows calculating the control reproduction number for the case that noncompliant individuals slightly change their behavior but to a lesser degree than compliant individuals (*a*_*c*_ < *a*_*n*_ < 1 and/or *s*_*c*_ < *s*_*n*_ < 1). Having assumed, however, that noncompliant individuals keep their pre-intervention behavior, we require that *a*_*n*_*s*_*n*_ = 1. Moreover, since we assumed the reproduction number to be reduced by a factor *r* < 1 if the compliance were 100*%* (meaning *ρ* = 1),^2^ we also require *r* = *a*_*c*_*s*_*c*_.

The simple formula for the control reproduction number is therefore as given in (5),

$$R_{c}=\left(r\rho +1-\rho \right)R_{0}=\left(1-\left(1-r\right)\rho \right)R_{0}.$$

The controlled reproduction number can be hence obtained reducing $R_{0}$ by a fraction (1 − *r*)*ρ*. The latter corresponds to the practical efficacy of control measures, being the product of the fraction effective contacts to be reduced by the measures and the compliance level.

If we drop the assumption on homogeneous detection (that is we allow *τ*_*c*_ ≠ *τ*_*n*_ and *η*_*c*_ ≠ *η*_*n*_), then the next generation matrix is calculated in a completely similar fashion:

$$FV^{-1}\;=\;\left(\begin{array}{ccc} A_{c}a_{c}s_{c}\rho & A_{n}a_{n}s_{c}\rho & *\ldots *\\ A_{c}a_{c}s_{n}\left(1-\rho \right) & A_{n}a_{n}s_{n}\left(1-\rho \right) & *\ldots *\\ 0 & 0 & 0\ldots 0\\ \vdots & \vdots & \vdots \end{array}\right),$$

with the abbreviation

$$A_{m}=\frac{\mu_{I}\left(\tau_{m}\gamma_{U}+\eta_{m}\right)}{\left(\gamma_{U}+\eta_{m}\right)\gamma_{I}}+\frac{1-\tau_{m}}{\gamma_{U}+\eta_{m}}+\frac{\mu_{E}}{\gamma_{E}},\;\;\;\;\;\;\;\;m\in \left\{c,n\right\}.$$

The eigenvalues are again 0 and

$$R_{c}=\beta_{0}\left(A_{c}a_{c}s_{c}\rho -A_{n}a_{n}s_{n}\left(1-\rho \right)\right).$$

Assuming again the behavior of the noncompliant population to be unaffected by the intervention, we obtain $\beta_{0}A_{n}=R_{0}$ and *a*_*n*_*s*_*n*_ = 1, leading to

$$R_{c}=\left(\frac{A_{c}}{A_{n}}r\rho -\left(1-\rho \right)\right)R_{0}.$$

The ratio $\frac{A_{c}}{A_{n}}$ depends on the particular values of the parameters involved, but the important message is that $R_{c}$ does not depend on whether the reduction in transmission is mediated by infectious individuals being cautious to not spread the virus or by susceptible individuals being cautious not to catch the virus. This will clearly be different once we are sufficiently far into the epidemic, i.e., sufficiently far away from the DFE. Since it should be expected that noncompliant individuals make up a larger share of the infectious population than their share in the total population is, while compliant individuals will more likely remain susceptible, the reduction *a*_*c*_ in spreading activity will play a less significant role compared to the reduction *s*_*c*_ in susceptibility.

## Appendix B Derivation of the correction factor of detection rates

To motivate the shape of the correction factor for the detection rate, *η*, given in (6) we consider a minimal model for an infectious disease with underascertainment. To this end, we track *S*usceptible, *U*ndetected infectious, detected *I*nfectious, and *R*emoved/recovered individuals. Moreover, we consider the population of available *T*ests.^3^ These are supplied at a given rate *σ*_+_ and are depleted by being applied to individuals who are tested. Clearly, not all tested individuals are infectious, hence tests are governed by the following quasi reactions:

$$\emptyset \overset{\sigma_{+}}{\to }T,T+S\overset{\sigma_{S}}{\to }S,T+U\overset{\sigma_{U}}{\to }I,\;T+I\overset{\sigma_{I}}{\to }I,T+R\overset{\sigma_{R}}{\to }R,$$

the first reaction describing the constant supply and the other reactions describing the pairing of a test with an individual (the person being tested). The test results allows only undetected individuals to change their state (from *U* to *I*), whereas leaves all other compartments unchanged. The resulting system of equations reads

$$\begin{array}{ll} \dot{T} & =\;\sigma_{+}-\left(\sigma_{S}S+\sigma_{U}U+\sigma_{I}I+\sigma_{R}R\right)T\\ \dot{S} & =\;-\left(\beta_{U}U+\beta_{I}I\right)S\\ \dot{U} & =\;\left(\beta_{U}U+\beta_{I}I\right)S-\gamma_{U}U-\sigma_{U}UT\\ \dot{I} & =\;\sigma_{U}UT-\gamma_{I}I\\ \dot{R} & =\;\gamma_{U}U+\gamma_{I}I. \end{array}$$

It now makes sense to choose *σ*_+_ and the *σ*_*X*_ sufficiently large to ensure that *T* is small. This reflects the fact that testing capacity cannot be stored since the limiting factor is laboratory time. Increasing testing capacity would result in larger *σ*_+_ and would allow for larger *σ*_*X*_, that is, a higher coverage, but should not result in large residual values of *T*.

This motivates the idea of viewing the test population as a fast variable, setting it to a quasi steady state

$$\dot{T}\approx 0\;\Rightarrow \;T\approx \frac{\sigma_{+}}{\sigma_{S}S+\sigma_{U}U+\sigma_{I}I+\sigma_{R}R}$$

and writing the only relevant testing term as

$$\sigma_{U}UT\;\approx \;\sigma_{U}\frac{\sigma_{+}}{\sigma_{S}S+\sigma_{U}U+\sigma_{I}I+\sigma_{R}R}\;U\;=\;\frac{\sigma_{+}}{K}\;\frac{K}{K+U}\;U$$

where we put

$$K=\frac{\sigma_{S}}{\sigma_{U}}S+\frac{\sigma_{I}}{\sigma_{U}}I+\frac{\sigma_{R}}{\sigma_{U}}R.$$

With the notation

$$\overset{-}{\eta }≔\frac{\sigma_{+}}{K}$$

we recover an analogue of relation (6). There are several assumptions at play in this discussion. First, we assume that *σ*_*U*_ is significantly larger than *σ*_*S*_, *σ*_*I*_, and *σ*_*R*_, meaning that for any given individual the chance of being tested is much larger if this individual is indeed infected. This chance may be influenced by the individual showing symptoms or having been in close contact to a confirmed case. While the derivation above remains valid for *σ*_*U*_ being similar to *σ*_*S*_, *σ*_*I*_, and *σ*_*R*_ and even if all *σ*_*X*_ were the same, which would be the case for random screening, the saturation effect would become less relevant. Indeed, if *σ*_*U*_ = *σ*_*S*_ = *σ*_*I*_ = *σ*_*R*_ then *K* = *S* + *I* + *R* and *σ*_*U*_*UT* ≈ *σ*_+_*U*/*N* with the constant population size *N*. Moreover, for small *σ*_*U*_, an unrealistically high number of tests would be required to reach a meaningful detection ratio at small prevalence. Considering again the case of random screening, to achieve a given detection ratio, this very fraction of individuals would have to be tested over the average infectious period which is about one week. For the sake of the argument, we may even assume *σ*_*I*_ = 0 since an already detected individual need not be detected again. We also assume the term *K* to be approximately constant. This is true if either the susceptible population is much larger than all other populations and *σ*_*S*_ is not much smaller than *σ*_*R*_, or if the rates *σ*_*S*_ and *σ*_*R*_ are approximately the same and *S* + *R* ≫ *I*. Both cases are plausible: If the detection ratio is high, most cases are detected and there are few recovered individuals who were not detected. Given the official COVID-19 data for Germany in the fall of 2020 (Robert Koch Institute Coronavirus Disease 2019 (COVID-19), 2019), this would mean the large majority of the population is still susceptible. If on the other hand, the detection ratio is low, there may be many recovered individuals who were never detected, but from the point of view of a test, these individuals are in no way different from susceptibles, and it should be expected that *σ*_*S*_ ≈ *σ*_*R*_.

In fact, simulating the above system (results not shown here) for *σ*_*S*_ = *σ*_*I*_ = *σ*_*R*_ and, say, *σ*_*U*_ = 20*σ*_*S*_, reveals that for most parameters, there is barely a difference between this system and the slow system

$$\begin{array}{ll} \dot{S} & =\;-\left(\beta_{U}U+\beta_{I}I\right)S\\ \dot{U} & =\;\left(\beta_{U}U+\beta_{I}I\right)S-\gamma_{U}U-\eta U\\ \dot{I} & =\;\eta U-\gamma_{I}I\\ \dot{R} & =\;\gamma_{U}U+\gamma_{I}I, \end{array}$$

with

$$\eta =\overset{-}{\eta }\frac{K}{K+U},$$

and *K* assumed to be constant. In contrast, the same system with constant $\eta =\overset{-}{\eta }$ shows significantly higher reported case numbers as soon as the prevalence increases. Moreover, if *β*_*I*_ is significantly smaller than *β*_*U*_ (as one would expect if detected cases are quarantined) the model with constant *η* predicts a slower progression of the disease than both the model with test population and the one with *U*-dependent detection rate.

## Appendix C Daily incidence in limit cases (cf. Fig. 6)

In Fig. 6a, we show the reductions of transmission required to achieve the goals of

(*i*) reducing within five weeks the incidence to 25*%* or 50*%* of the value at intervention, or
(*ii*) preventing the incidence from significantly rising at any time within weeks 2 through 5 after intervention,

introduced in subsection 3.1. Here we show the actual course of the incidence if contacts are reduced by these exact threshold levels. The corresponding curves are shown in Fig. A1 for selected prescribed compliance levels and under the assumptions of both late and early detection.

As already obvious from Fig. 6a for late detection (the curves for early detection being very similar), neither goal (*i*) or (*ii*) can be achieved at 30*%* compliance, and even without any transmission from or to compliant individuals (dashed curves, upper left panels in Fig. A1(a)-(b)) the incidence will rise after possibly briefly falling. At 40*%* compliance, stagnating incidence levels can be achieved but transmission among compliant individuals must be very small (blue curves, upper right panels in Fig. A1(a)–(b)). As compliance rises to 50*%*, a reduction of the incidence to half the value at intervention is possible within five weeks, and finally, if at least 60*%* of the population adhere to the contact reductions, either goal can be achieved, though clearly at different contact reductions. For compliance levels higher than 60*%* the incidence curves achieved by the threshold transmission reductions do not change significantly. This means that there is no big difference in the outcome upon very high compliance with moderately effective reduction measures, and moderate compliance with very strong reductions in transmission.

Another effect, already alluded to in the discussion of scenario 2′, becomes apparent from Fig. A1. In the case of late detection, the threshold contact reduction that is necessary for stagnation results in the incidence leveling out close to the maximum value. The latter is reached when the full effect of the intervention starts showing in the reported case numbers. Quite differently, for the case of early detection, the same threshold value leads to a significant decline in newly detected cases before a plateau well below the maximum incidence is reached. This can potentially lead to a deceptive situation where the rapidly falling incidence may make the contact reductions to appear way more effective than they are, and consequently reduce the sense of urgency among the population. If this in turn leads to a lower level of compliance, the incidence instead of falling further or stabilizing at a level slightly below the maximum may soon start rising again. However, as will become apparent from the following discussion, the incidence may start rising after a brief decrease even in the absence of any further changes of the transmission rates.

This effect is not caused by reporting delays or underascertainment but is inherent to the transmission dynamics as is apparent from the fact that the true incidence of new infections shows the same behavior. For the sake of simplicity, we shall illustrate here the argument on the basis of the SIR model, neglecting both the latent period and possible under ascertainment,

(A1) $$\begin{array}{ll} {\dot{S}}_{c} & =\;-\left(\beta_{cc}I_{c}+\beta_{nc}I_{n}\right)S_{c}\\ {\dot{S}}_{n} & =\;-\left(\beta_{cn}I_{c}+\beta_{nn}I_{n}\right)S_{n}\\ {\dot{I}}_{c} & =\;\left(\beta_{cc}I_{c}+\beta_{nc}I_{n}\right)S_{c}-\gamma I_{c}\\ {\dot{I}}_{n} & =\;\left(\beta_{cn}I_{c}+\beta_{nn}I_{n}\right)S_{n}-\gamma I_{n}, \end{array}$$

with the removed compartments, *R*_*m*_ = *N*_*m*_ − *S*_*m*_ − *I*_*m*_, *m* ∈{*c*, *n*}.

In keeping with our previous notation, we assume a fixed transmission parameter *β*_0_ that is constant among all pairs (*I*_*m*_, *S*_*k*_), and describes transmission before the intervention. After interventions, the transmission parameters are again given by *β*_*mk*_ = *a*_*m*_*s*_*k*_*β*_0_, and we recall that *a*_*n*_ = *s*_*n*_ = 1. Instead of calculating next generation matrices we now linearize system (A1) about the disease free equilibrium (*ρN*, (1 − *ρ*)*N*, 0, 0), *ρ* again denoting the compliant fraction of the population. This leads to

$$\frac{d}{dt}\left(\begin{array}{c} S_{c}\\ S_{n}\\ I_{c}\\ I_{n} \end{array}\right)\approx \left(\begin{array}{cccc} 0 & 0 & -\beta_{0}a_{c}s_{c}\rho N & -\beta_{0}s_{c}\rho N\\ 0 & 0 & -\beta_{0}a_{c}\left(1-\rho \right)N & -\beta_{0}\left(1-\rho \right)N\\ 0 & 0 & \beta_{0}a_{c}s_{c}\rho N-\gamma & \beta_{0}s_{c}\rho N\\ 0 & 0 & \beta_{0}a_{c}\left(1-\rho \right)N & \beta_{0}\left(1-\rho \right)N-\gamma \end{array}\right)\left(\begin{array}{c} S_{c}\\ S_{n}\\ I_{c}\\ I_{n} \end{array}\right).$$

The constant population size allows us to abbreviate *β* = *β*_0_*N*, and we only have to investigate the lower right submatrix

$$J=\left(\begin{array}{cc} a_{c}s_{c}\rho \beta -\gamma & s_{c}\rho \beta \\ a_{c}\left(1-\rho \right)\beta & \left(1-\rho \right)\beta -\gamma \end{array}\right).$$

Its eigenvalues are

$$\lambda_{1}=\beta \left(a_{c}s_{c}\rho +\left(1-\rho \right)\right)-\gamma \;\;\;\;\;\;\;\text{ and }\;\;\;\;\;\;\;\lambda_{2}=-\gamma <\lambda_{1},$$

and obviously the sign of *λ*_1_ determines the stability of the DFE. There is nothing new here, the same information was obtained by calculating the dominant eigenvalue of the next generation matrix that would have been

$$R_{c}=\frac{\beta \left(a_{c}s_{c}\rho +\left(1-\rho \right)\right)}{\gamma }.$$

However, linearizing about the DFE allows us to investigate the corresponding eigenvectors

$$\xi_{1}={\left(s_{c}\rho ,1-\rho \right)}^{T}\;\;\;\;\;\;\;\text{ and }\;\;\;\;\;\;\;\xi_{2}={\left(1,-a_{c}\right)}^{T}$$

of *J*, which tell us how the infections have to be distributed among compliant and noncompliant individuals in order to create the maximal number of secondary infections.

Before intervention, there is no difference between compliant and noncompliant individuals and *a*_*c*_ = *s*_*c*_ = 1. Having assumed $R_{0}>1$ means that the leading eigenvalue before intervention is $\lambda_{1}^{0}>0$, hence the system will have evolved to a situation where ${\left(I_{c},I_{n}\right)}^{T}$ is proportional to $\xi_{1}^{0}={\left(\rho ,1-\rho \right)}^{T}$. At the time of intervention, the system is still close to the DFE, *S*_*c*_ + *S*_*n*_ still making up the vast majority of the population. Let us now assume that the control reproduction number after intervention is $R_{c}⪆1$, meaning that the controlled eigenvalue $\lambda_{1}^{c}⪆0$ is still positive but small. However, the distribution of infectious individuals is no longer aligned with the corresponding eigenvector $\xi_{1}^{c}$ but still with $\xi_{1}^{0}$. Hence, the component of the solution aligned with $\xi_{1}^{c}$ will slowly increase at rate $\lambda_{1}^{c}$ while the component aligned with $\xi_{2}^{c}$ will decrease at rate *λ*_2_ = −*γ*. If $\lambda_{1}^{c}$ is sufficiently small, this leads to an overall decrease of the number of infectious individuals until the solution is sufficiently aligned to $\xi_{1}^{c}$ and the component proportional to $\xi_{2}^{c}$ becomes negligible.

In order to illustrate this effect in a numerical example, let us work with our previous assumption that $R_{0}=1.5$, meaning in this simplified setting that *β* = 1.5*γ* or $\lambda_{1}^{0}=\beta -\gamma =0.5\gamma$. This allows us to write

$$\lambda_{1}=\gamma \left(\frac{3}{2}\left(a_{c}s_{c}\rho +\left(1-\rho \right)\right)-1\right)=\frac{\gamma }{2}\left(1-3\left(1-a_{c}s_{c}\right)\rho \right)=\lambda_{1}^{0}\left(1-3\left(1-r\right)\rho \right),$$

where *r* = *a*_*c*_*s*_*c*_. Just as we calculated the control reproduction number as a multiple of $R_{0}$, we can now calculate $\lambda_{1}^{c}$ as a multiple of $\lambda_{1}^{0}$ by a factor depending solely on the transmission reduction among compliant individuals, *r*, and the compliant fraction *ρ* of the population. It should not come as a surprise that Fig. A2a looks rather similar to Fig. 6b.

As the eigenvector $\xi_{1}^{c}$ depends on *s*_*c*_ rather than *r* as a whole, we need to make a choice on how to distribute the contribution to *r* among reduced effective infectiousness, *a*_*c*_, and reduced effective susceptibility, *s*_*c*_, of compliant individuals. For the sake of showing the eigenvector in Fig. A2b, we choose $a_{c}=s_{c}=\sqrt{r}$, meaning that both effects contribute equally. Doing so allows us to illustrate how strongly $\xi_{1}^{c}$ is shifted towards *I*_*n*_ as compared to $\xi_{1}^{0}$. We have chosen to fix *r* = 0.25 for figure *A*2*b*, and it should be obvious that the shift is stronger the smaller *r*, and hence *s*_*c*_, is. Indeed, Fig. A3 illustrates how the solution vector shifts toward the new eigenvector after the intervention.

In fact, the observed phenomenon is not exclusively due to partial noncompliance. We may consider the classical SIR model ($\dot{S}\;=\;-\beta IS,\;\dot{I}\;=\;\beta IS-\gamma I$) with reproduction number $R_{0}=\frac{\beta }{\gamma }>1$, and assume that at some point in time the contact rate *β* is reduced to *rβ* with 0 < *r* < 1. At the time of intervention, the incidence of new infections will instantaneously drop by the reduction factor *r*. However, let if the contact reduction is so weak that $R_{c}=r\frac{\beta }{\gamma }>1$, then the incidence will immediately start rising again. Adding a latency period and explicitly modeling detection will smooth this process but an intermittently decreasing incidence followed by another rise may still be visible. This reasoning shows that there is no need to assume a reaction of the population to falling incidence values in order to observe rebounding incidence numbers. Conversely, these arguments should also caution against relaxing contact restrictions too quickly as this could exacerbate the rebound effect by increasing $R_{c}$.

## Appendix D Model parameters

In the following list we summarize the model parameters relevant for the transmission dynamics of (1) and their values used for the simulations.

*N*: total population (assumed to be constant in good approximation)83, 000, 000approximately the population of Germany as of 2020
*β*_0_: global transmission rate0.225 d^−1^/N directly before the intervention; slightly smaller at the start of the simulated period as indicated at the beginning of Section 3fixed to achieve $R_{0}\approx 1.5$ given the other parameters
*μ*_*U*_: relative transmission rate for undetected infectious individuals 1 can always be fixed as 1 by appropriately choosing the common factor *β*_0_ which is exactly what we did
*μ*_*E*_: relative transmission rate for presymptomatic infectious individuals1.5 assures that $R_{E}$ contributes 35*%*–40*%* to $R_{0}$
*μ*_*I*_: relative transmission rate for detected infectious individuals 0.1 represents significantly decreased transmission due to (self) isolation
*γ*_*E*_: three times inverse of mean duration of incubation period 3/(5 *d*)corresponds to mean incubation period of five days
*γ*_*U*_: inverse of mean duration of symptomatic period1/(7 *d*)
*γ*_*I*_: inverse of mean duration of infectious period after detection 1/(7 *d*) same as *γ*_*U*_ in order to ensure that detection does not affect mean infectious period
$\overset{-}{\tau }$: maximal fraction of infectious individuals detected at symptom onset0.1
$\overset{-}{\eta }$: maximal detection rate from symptomatic stage *U* 0.6*γ*_*U*_chosen such that approximately 45*%* of infected individuals will be detected at low prevalence
*K*: prevalence of *undetected* infectious individuals at which the detection rates decrease to half their maximal value500, 000
